# Preoperative deep vein thrombosis in tibial plateau fractures: development and internal validation of an interpretable multivariable machine-learning diagnostic model

**DOI:** 10.3389/fmed.2026.1730477

**Published:** 2026-02-13

**Authors:** Dejun Cun, Junru Li, Paian He, Lin Zhou, Hang Dong, Feng Huang, Ziwei Jiang

**Affiliations:** 1 The First Clinical Medical College of Guangzhou University of Chinese Medicine, Guangzhou, China; 2 Department of Lower Limb Trauma Orthopedics, The First Affiliated Hospital of Guangzhou University of Traditional Chinese Medicine, Guangzhou, China

**Keywords:** D-dimer, deep vein thrombosis, diagnostic prediction, explainable machine learning (SHAP), machine learning, tibial plateau fracture

## Abstract

**Background:**

Preoperative deep vein thrombosis (DVT) is common in tibial plateau fractures (TPF), yet few tools target this window with calibration and clinical utility reporting.

**Methods:**

Single-center retrospective cohort (2019–2024) of adults undergoing surgery for isolated TPF. Outcome: duplex ultrasonography–confirmed DVT before initiation of therapeutic anticoagulation. Candidate predictors included demographics; injury features (Schatzker type/side, injury-to-surgery interval); and coagulation, inflammatory, and nutritional–immune indices. Features were selected by the intersection of LASSO and Boruta. Data were split 7:3 into training/validation; seven algorithms were tuned with 5-fold cross-validation. Validation assessed AUROC (95% confidence interval), Brier score, calibration, and decision-curve analysis (DCA). Model interpretability was assessed using SHAP (Shapley Additive Explanations).

**Results:**

Among 894 patients, 299 (33.4%) had preoperative DVT. Nine predictors were retained: D-dimer, age, erythrocyte sedimentation rate, prognostic nutritional index, C-reactive protein, lymphocyte count, Schatzker type, neutrophil count, and smoking. XGBoost performed best (AUROC 0.840, 95% confidence interval 0.790–0.884; accuracy 0.787; sensitivity 0.640; specificity 0.860; F1 score 0.667; Brier 0.149) and provided net clinical benefit on DCA. Tree-ensemble models showed training–validation performance gaps, indicating overfitting. SHAP ranked D-dimer and age as dominant with non-linear effects; higher C-reactive protein and erythrocyte sedimentation rate, lower prognostic nutritional index, advanced Schatzker types, and smoking increased risk.

**Conclusion:**

An interpretable XGBoost model based on routine preoperative variables identifies TPF patients at high risk of preoperative DVT and may guide ultrasound triage and perioperative management. External (multicenter and temporal) validation with recalibration and prospective impact assessment are required.

## Introduction

In patients with tibial plateau fractures (TPF), tissue injury from the intra-articular fracture, immobilization, and a hypercoagulable state converge during the preoperative period, markedly increasing the risk of deep vein thrombosis (DVT). Prospective and retrospective cohorts using routine bilateral lower-limb ultrasonography typically report a preoperative DVT incidence of about 16%–24% in TPF; for example, Zhu et al. observed a 16.3% rate, predominantly distal, in a prospective cohort (1). Recent work also identified 23.6% “occult” preoperative DVT despite prophylactic anticoagulation (2), suggesting that the true burden may be underestimated. Conversely, both higher and lower rates have been reported (3, 4), reflecting variation in screening strategies, injury severity, ultrasound timing, and the use of routine prophylaxis, and indicating substantial heterogeneity. Preoperative DVT not only heightens peri-anesthetic and perioperative pulmonary embolism risk but also influences anticoagulation strategy, surgical timing, and procedure selection, thereby prolonging hospitalization and increasing costs. Early, accurate identification of high-risk individuals is therefore clinically essential.

Mechanistically, TPF embody Virchow’s triad: intra-articular fracture and subchondral bone injury cause endothelial disruption; post-injury pain and immobilization produce venous stasis; and the trauma stress response, tissue factor release, and systemic inflammation induce hypercoagulability. Multiple studies have identified observable preoperative DVT risk factors across clinical and laboratory domains, including older age, diabetes, smoking, obesity, longer injury-to-surgery delay, fracture classification, elevated coagulation indices such as D-dimer, abnormal inflammatory markers (CRP, ESR), dysregulated immune–inflammatory ratios (e.g., LMR, NLR, SII), and reduced nutritional–immune status (PNI) (5–7). These variables provide measurable biological foundations for prediction models centered on injury features, coagulation/inflammation, and timing.

Most research on DVT in orthopedic populations centers on hip fractures or the postoperative period, leaving limited evidence for risk prediction specifically in preoperative TPF. Prior models largely rely on traditional regression, which struggles to capture non-linear interactions and collinearity among multidomain features spanning inflammation, coagulation, and nutrition; reporting of calibration and clinical net benefit (DCA) has also been sparse. Recent studies applying machine learning (ML) to VTE/DVT prediction across hip, spine, and thoracic trauma cohorts show promising discrimination and generalizability (8), yet ML models tailored to preoperative DVT in TPF remain scarce. Comprehensive assessment that integrates discrimination, calibration, clinical utility, and interpretability is lacking, hindering clinical adoption. Here, using a single-center, consecutively enrolled TPF cohort, we integrate demographics, fracture characteristics (classification, laterality), imaging, and laboratory data to develop and internally validate an ML model for preoperative DVT. We compare multiple algorithms; evaluate discrimination using receiver operating characteristic curves and precision–recall curves, calibration using Brier score and calibration plots, and clinical net benefit using decision-curve analysis (DCA); and use SHAP to provide both global and individual-level interpretability. Our goal is to offer a quantitative tool for individualized perioperative screening and intervention. The TRIPOD+ AI guideline underscores the need for standardized reporting of regression- and ML-based prediction models and provides methodological guidance for such studies (9).

## Materials and methods

### Patient cohort

**FIGURE 1:**
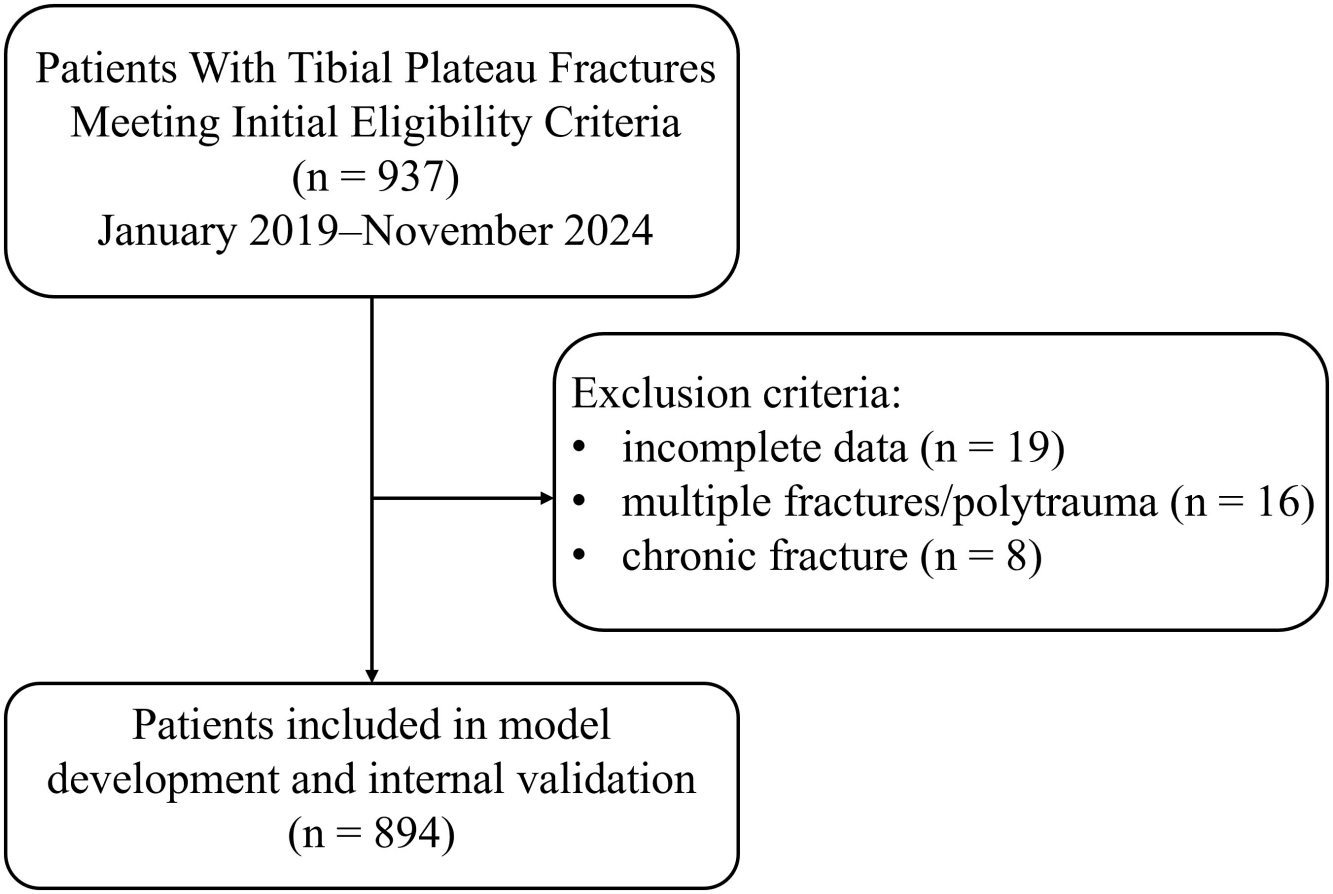
Patient selection flowchart for tibial plateau fractures.

We conducted a single-center retrospective cohort including consecutive adults (≥18 years) admitted for TPF and subsequently undergoing surgery for isolated TPF between January 2019 and November 2024. Inclusion criteria were radiographic (X-ray/CT) confirmation of TPF; age ≥ 18 years; an acute fracture (injury-to-assessment ≤ 21 days); first hospitalization for the index injury; availability of preoperative clinical data, including lower-limb venous duplex ultrasonography performed before initiation of therapeutic-dose anticoagulation; and collection of all candidate predictors preoperatively. Exclusion criteria were concomitant long-bone or pelvic/acetabular fractures or polytrauma (small peri-knee avulsions, such as fibular head avulsion, were not deemed polytrauma but were recorded); old fractures (>21 days from injury); pathologic or metastatic fractures; therapeutic-dose anticoagulation initiated before preoperative ultrasonography; pregnancy or ≤6 weeks postpartum; prior venous thromboembolism or known hereditary/acquired thrombophilia; and incomplete clinical data, including missing outcomes or non-imputable absence of key predictors. During the study period, 937 patients met the initial screening criteria; 19 were excluded for incomplete data, 16 for multiple fractures/polytrauma, and eight for old fractures, leaving 894 patients for model development and internal validation (Figure 1).

### Methods and variables

Preoperative clinical and laboratory data were extracted from the electronic medical record and Laboratory Information System. All candidate predictors were collected before surgery and prior to initiation of therapeutic-dose anticoagulation. Variables included: (1) demographics/clinical course: sex, age, body mass index (BMI), season of injury (March–May, June–August, September–November, December–February), injury-to-surgery interval (days), Schatzker type, laterality (left/right/bilateral), comorbidities (hypertension, diabetes, coronary artery disease), alcohol use, and smoking; (2) first preoperative complete blood count: white blood cell count (WBC), neutrophil count (NEU), lymphocyte count (LYM), monocyte count (MONO), platelet count (PLT), mean platelet volume (MPV), and hemoglobin (HGB); (3) derived immune–inflammatory indices: NLR = NEU/LYM, PLR = PLT/LYM, LMR = LYM/MONO, SII = PLT × NEU/LYM, SIRI = NEU × MONO/LYM, and PNI was calculated as serum albumin + 5 × LYM (albumin from the first preoperative chemistry); (4) coagulation markers: fibrinogen (FIB), activated partial thromboplastin time (APTT), prothrombin time (PT), and D-dimer; and (5) additional inflammatory markers: C-reactive protein (CRP) and erythrocyte sedimentation rate (ESR). Laboratory values were sourced from the Laboratory Information System; when multiple same-day tests were available, we selected the preoperative result closest in time to the lower-limb venous color Doppler ultrasonography. Units and data entries were checked for plausibility and corrected as needed. Right-skewed variables were log-transformed when appropriate and modeled as continuous. The primary outcome was preoperative DVT confirmed by lower-limb color Doppler duplex ultrasonography; patients were classified into thrombosis and non-thrombosis groups for comparisons and model development.

### Model development and assessment

All machine-learning analyses were performed in Python 3.10. We randomly split the cohort 70:30 using stratified sampling to preserve event prevalence in the training and validation sets. The training set was used for model fitting and hyperparameter tuning; the held-out validation set was reserved for estimating generalization. We evaluated seven common binary classifiers: decision tree (DT), random forest (RF), XGBoost, support vector machine (SVM), artificial neural network (ANN), LightGBM, and logistic regression (LR). Hyperparameters were optimized on the training set with grid search and 5-fold cross-validation, using AUROC as the objective. Candidate ranges were chosen from prior experience and interpretability: for example, tree depth and minimum samples per split for decision trees; learning rate and number of estimators for boosting models; kernel type and regularization for SVM; hidden units and epochs for ANN; and penalty type and strength for logistic regression. The validation set was never used during tuning. Performance was reported for both the training and validation sets. We presented confusion matrices and calculated accuracy, positive predictive value, sensitivity (recall), specificity, F1 score, negative predictive value, and AUROC with 95% confidence intervals. ROC curves, calibration plots, and decision-curve analysis (DCA) were used to assess discrimination, calibration, and net clinical benefit. To enhance interpretability, we applied SHAP to the best-performing model, providing global feature importance and beeswarm/dependence plots to characterize overall effects, as well as local explanations for individual patients. This approach improves transparency and supports clinical understanding of model decisions.

### Descriptive statistics

All statistical analyses were performed in R (v4.5.1; R Foundation for Statistical Computing, Vienna, Austria). Continuous variables are reported as mean ± standard deviation. Between-group comparisons used the independent-samples *t*-test or the Mann–Whitney U test according to normality assessed by the Shapiro–Wilk test. Categorical variables are summarized as counts (%) and compared with the χ^2^ test or Fisher’s exact test, as appropriate. Two-sided *p* < 0.05 was considered statistically significant. Missing data were addressed with multiple imputation using the mice package (R 4.5.1) under a prespecified protocol with post-imputation consistency checks. Records with missing outcomes or non-imputable key predictors were excluded during screening.

### Ethical aspects

This retrospective observational study was approved by the Ethics Committee of the First Affiliated Hospital of Guangzhou University of Chinese Medicine (approval No. JY2025-116; 15 August 2025). Because only existing electronic medical records and laboratory data were used, with no additional interventions or patient contact, the committee waived written informed consent. The study complied with the Declaration of Helsinki; all data were de-identified before analysis and are reported in aggregate.

## Results

### Baseline characteristics of the study cohort

Table 1 summarizes between-group differences. Among 894 patients with tibial plateau fractures, 299 (33.4%) had preoperative DVT. Compared with the non-DVT group, patients with DVT were older and had higher BMI (both *p* < 0.05). Smoking, hypertension, diabetes, and coronary artery disease were more prevalent (all *p* < 0.05), whereas sex, fracture laterality, and the injury-to-surgery interval did not differ significantly. Fracture type distribution also differed, with higher proportions of Schatzker II and VI in the DVT group (*p* < 0.01). In laboratory tests, PLT, MPV, and HGB were lower (*p* < 0.05), while WBC and NEU counts were higher (*p* < 0.01). Coagulation and inflammatory markers were elevated in the DVT group, including D-dimer and FIB, as well as CRP and ESR. Derived indices (NLR, SII, SIRI, PNI) also differed between groups (*p* < 0.05).

**TABLE 1:** Demographic and clinical characteristics of the study population.

Variables	Total (*n* = 894)	No DVT (*n* = 595)	DVT (*n* = 299)	*P*-value
Age (years)	48.32 ± 16.51	43.63 ± 15.12	57.65 ± 15.14	<0.01
Sex (male, %)	505 (56.49)	344 (57.82)	161 (53.85)	0.26
BMI (kg/m^2^)	23.80 ± 3.05	23.65 ± 3.13	24.09 ± 2.85	0.04
Fracture onset (%)				0.02
March–May	186 (20.81)	118 (19.83)	68 (22.74)	–
June–August	292 (32.66)	191 (32.10)	101 (33.78)	–
September–November	144 (16.11)	112 (18.82)	32 (10.70)	–
December–February	186 (20.81)	118 (19.83)	68 (22.74)	–
Smoking (%)	225 (25.17)	132 (22.18)	93 (31.10)	<0.01
Hypertension (%)	165 (18.46)	88 (14.79)	77 (25.75)	<0.01
Diabetes (%)	86 (9.62)	38 (6.39)	48 (16.05)	<0.01
Drink (%)	293 (32.77)	182 (30.59)	111 (37.12)	0.05
History of CAD (%)	28 (3.13)	9 (1.51)	19 (6.35)	<0.01
Side (%)				0.36
Left	493 (55.15)	338 (56.81)	155 (51.84)	–
Right	386 (43.18)	247 (41.51)	139 (46.49)	–
Bilateral	15 (1.68)	10 (1.68)	5 (1.67)	–
Schatzker type (%)				<0.01
I	68 (7.61)	48 (8.07)	20 (6.69)	–
II	205 (22.93)	144 (24.20)	61 (20.40)	–
III	121 (13.53)	73 (12.27)	48 (16.05)	–
IV	235 (26.29)	186 (31.26)	49 (16.39)	–
V	81 (9.06)	63 (10.59)	18 (6.02)	–
VI	184 (20.58)	81 (13.61)	103 (34.45)	–
Interval (days)	7.92 ± 3.48	7.92 ± 3.48	7.92 ± 3.50	0.99
DDi (mg/L)	6.45 ± 8.08	4.44 ± 5.90	10.44 ± 10.10	<0.01
APTT (s)	24.98 ± 4.10	25.13 ± 4.16	24.69 ± 3.97	0.13
PT (s)	10.97 ± 0.91	10.95 ± 0.89	11.00 ± 0.93	0.45
FIB (g/L)	3.66 ± 1.39	3.49 ± 1.29	4.01 ± 1.52	<0.01
WBC (10^9^/L)	8.67 ± 2.69	8.46 ± 2.65	9.09 ± 2.72	<0.01
PLT (10^9^/L)	244.14 ± 83.69	247.95 ± 83.26	236.57 ± 84.16	0.01
NEU (10^9^/L)	6.08 ± 2.48	5.83 ± 2.40	6.60 ± 2.58	<0.01
MONO (10^9^/L)	0.64 ± 0.33	0.64 ± 0.36	0.64 ± 0.26	0.76
LYM (10^9^/L)	1.80 ± 0.68	1.80 ± 0.66	1.82 ± 0.73	0.72
MPV (fL)	9.31 ± 0.99	9.36 ± 1.02	9.21 ± 0.93	0.03
HGB (g/L)	125.53 ± 20.27	128.77 ± 19.53	119.09 ± 20.23	<0.01
CRP (mg/L)	33.57 ± 37.90	28.60 ± 36.44	43.48 ± 38.86	<0.01
ESR (mm/h)	22.48 ± 20.47	18.19 ± 17.14	31.01 ± 23.66	<0.01
NLR	4.16 ± 3.47	3.85 ± 3.35	4.76 ± 3.62	<0.01
PLR	153.30 ± 77.28	149.98 ± 70.84	159.90 ± 88.47	0.07
LMR	3.24 ± 1.67	3.26 ± 1.68	3.21 ± 1.67	0.73
SII	973.14 ± 750.03	919.34 ± 700.19	1080.20 ± 831.41	<0.01
SIRI	96.58 ± 84.20	92.20 ± 65.29	105.30 ± 112.41	0.03
PNI	41.34 ± 3.79	42.07 ± 3.10	39.88 ± 4.56	<0.01

### Feature selection: LASSO and Boruta

**FIGURE 2:**
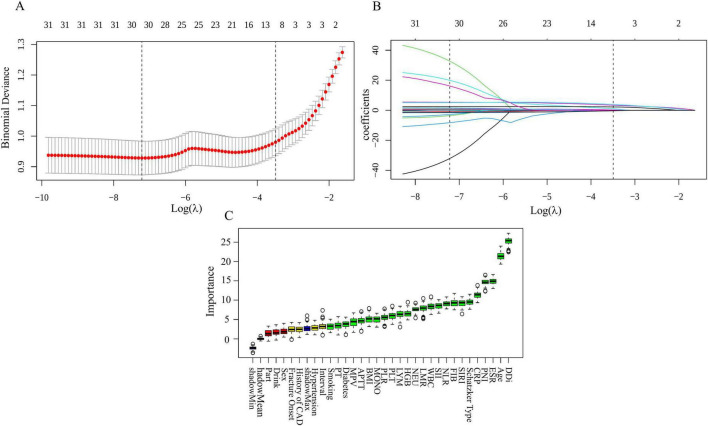
Feature selection by LASSO and Boruta. **(A)** A total of 10-fold cross-validated LASSO. **(B)** LASSO coefficient paths across log(λ). **(C)** Boruta feature importance ranking.

Feature selection used two complementary approaches: LASSO and Boruta. LASSO identified nine candidate predictors for preoperative DVT in TPF—D-dimer, age, ESR, PNI, CRP, LYM, Schatzker type, NEU, and smoking (Figures 2A,B). Boruta corroborated these variables and, in addition, flagged hypertension, diabetes, injury-to-surgery interval, BMI, WBC, PLT, MONO, MPV, HGB, APTT, PT, FIB, NLR, PLR, LMR, SII, and SIRI as associated features (Figure 2C). To ensure robustness, we retained the intersection of LASSO and Boruta—D-dimer, age, ESR, PNI, CRP, LYM, Schatzker type, NEU, and smoking—as the final predictor set. This overlap offers both statistical stability and clinical plausibility. LASSO (L1 regularization) selects the most predictive variables, whereas Boruta (random-forest–based importance with shadow features) tests relevance; using their intersection improves accuracy and consistency. These variables were then used as model inputs. Before feature selection and model fitting, we inspected pairwise correlations among continuous candidate predictors using a Pearson correlation matrix and heatmap (Supplementary Figure 2). For any pair with an absolute correlation coefficient ≥ 0.7, we retained the clinically more informative or widely used variable and excluded its counterpart to limit redundancy due to collinearity. The nine predictors that entered the final XGBoost model did not exceed this prespecified threshold.

### Model development and evaluation

**FIGURE 3:**
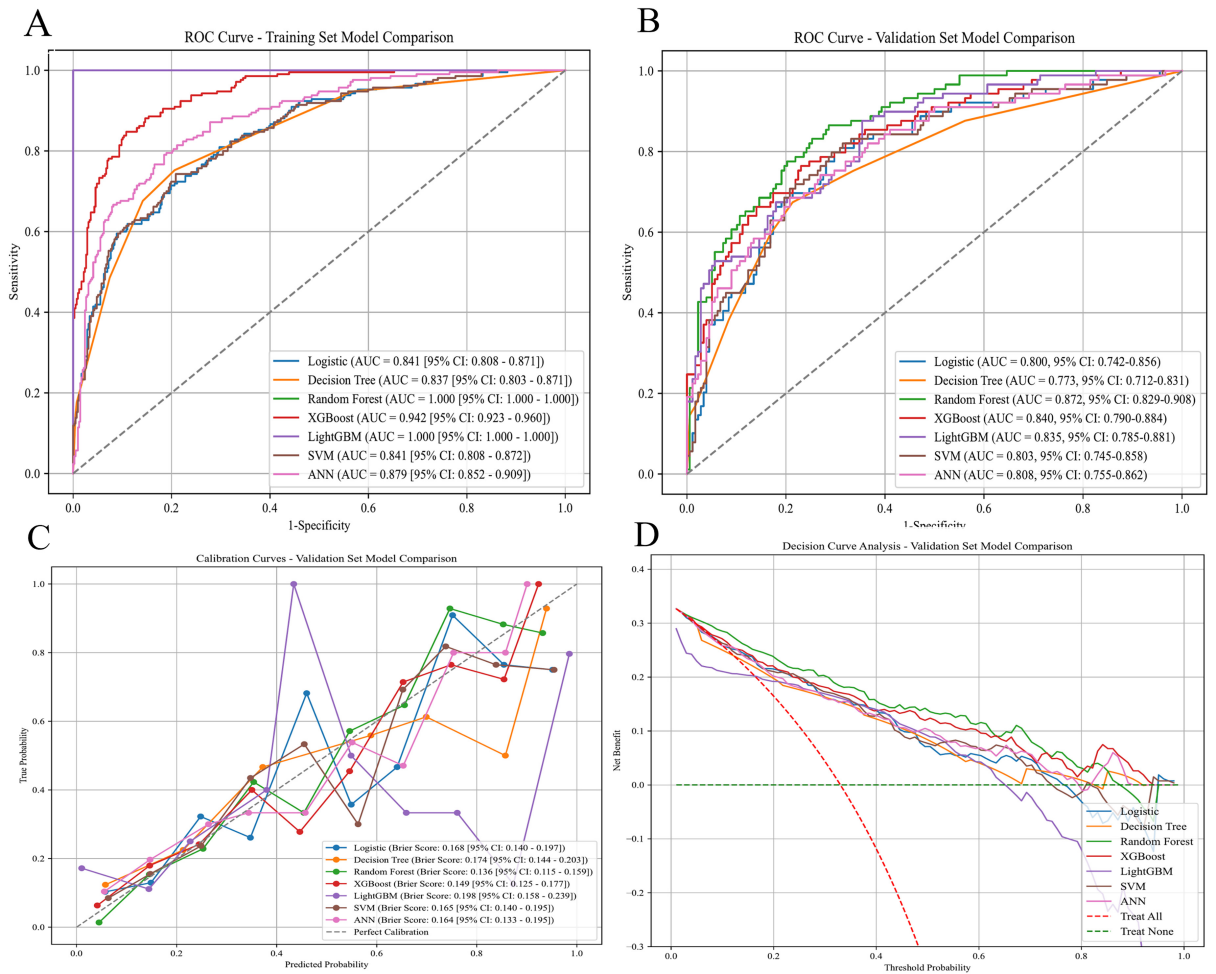
Comprehensive evaluation of machine-learning models. **(A)** ROC curves with AUC in the training set. **(B)** ROC curves with AUC in the validation set. **(C)** Calibration curves with Brier scores in the validation set. **(D)** DCA in the validation set. ROC, receiver operating characteristic; AUC, area under the ROC curve; DCA, decision-curve analysis; LR, logistic regression; DT, decision tree; RF, random forest; XGBoost, extreme gradient boosting; LightGBM, Light Gradient Boosting Machine; SVM, support vector machine; ANN, artificial neural network.

We developed models using seven algorithms: DT, RF, XGBoost, LightGBM, ANN, SVM, and LR. Discrimination, calibration, and clinical net benefit were displayed via ROC curves, calibration plots, and DCA (Figure 3) and quantified by AUROC, accuracy, sensitivity, specificity, precision, positive predictive value, negative predictive value, and F1 score (Table 2). All models were tuned in the training set with 5-fold cross-validation; the validation set was used only for independent evaluation. On the validation set, XGBoost achieved the best overall balance (AUROC 0.840, 95% confidence interval 0.790–0.884; accuracy 78.7%; sensitivity 64.0%; precision 69.5%; specificity 86.0%; F1 score 66.7%) and maintained strong discrimination in the training set (AUROC 0.942, 95% confidence interval 0.923–0.960). RF and LightGBM reached AUROC = 1.000 in the training set (several LightGBM metrics were also 1.000) but showed attenuation on validation, with RF yielding the highest validation AUROC (0.872). The training–validation gaps in these tree-ensemble models indicate some overfitting. We therefore selected XGBoost as the primary model based on its stable discrimination, calibration, and DCA performance; the remaining models (DT, SVM, LR, ANN) exhibited moderate discrimination but overall underperformed XGBoost.

**TABLE 2:** Predictive performance of candidate models in the validation set.

Model	AUC (95% CI)	Accuracy	Sensitivity	Specificity	PPV	NPV	F1 score	Brier score
RF	0.872 (0.829–0.908)	0.809	0.640	0.893	0.750	0.832	0.691	0.136
XGBoost	0.840 (0.790–0.884)	0.787	0.640	0.860	0.695	0.827	0.667	0.149
LightGBM	0.835 (0.785–0.881)	0.760	0.607	0.837	0.651	0.810	0.628	0.198
ANN	0.808 (0.755–0.862)	0.772	0.584	0.865	0.684	0.806	0.630	0.164
SVM	0.803 (0.745–0.858)	0.745	0.472	0.882	0.667	0.770	0.553	0.165
LR	0.800 (0.742–0.856)	0.738	0.494	0.860	0.638	0.773	0.557	0.168
DT	0.773 (0.712–0.831)	0.753	0.596	0.831	0.639	0.804	0.616	0.174

LR, logistic regression; DT, decision tree; RF, random forest; SVM, support vector machine; ANN, artificial neural network. Performance metrics were computed on the independent validation set. AUC with 95% CIs was estimated using the DeLong method. Threshold-based metrics (Accuracy, Sensitivity, Specificity, PPV, NPV, F1) used a probability cutoff selected by maximizing Youden’s J within training cross-validation and then applied to the validation set. Brier score was calculated from predicted probabilities on the validation set.

### Interpretability and application of the model

**FIGURE 4:**
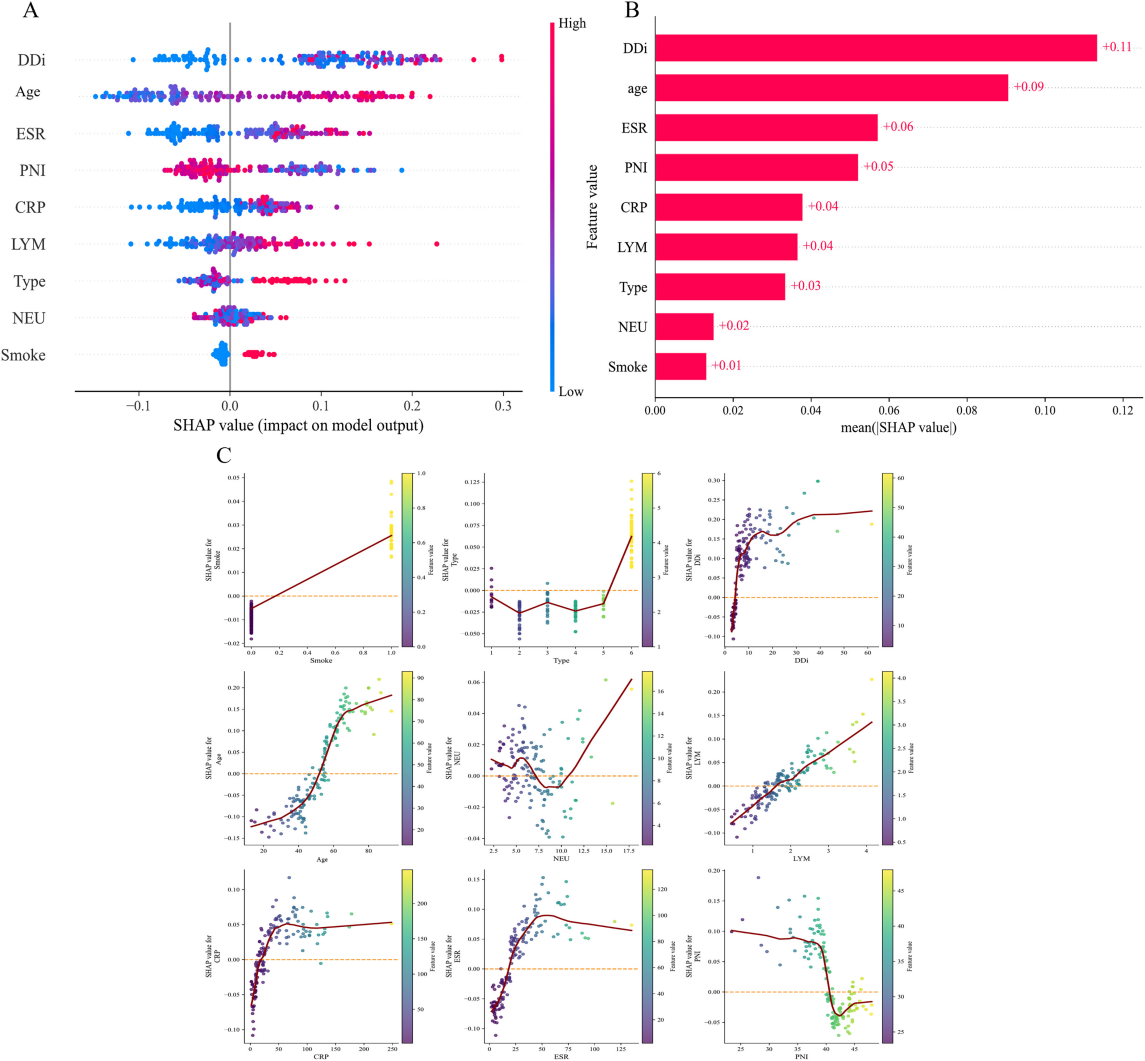
Model interpretability with Shapley Additive Explanations (SHAP) extreme gradient boosting (XGBoost). **(A)** SHAP beeswarm plot: distribution of SHAP values for each predictor; color encodes feature value (blue, low; red, high). **(B)** SHAP feature-importance bar plot: predictors ranked by mean absolute SHAP value. **(C)** SHAP dependence plots for the nine predictors (points, individuals; solid curve, smoothed trend; orange dashed line, SHAP 0 reference): smoking status (0, no; 1, yes), Schatzker type (I–VI, ordinal), age, D-dimer, NEU, LYM, CRP, ESR, and PNI. NEU, neutrophil count; LYM, lymphocyte count; CRP, C-reactive protein; ESR, erythrocyte sedimentation rate; PNI, prognostic nutritional index.

Figure 4A shows SHAP summaries for the final model, quantifying each feature’s contribution to predicted preoperative DVT risk in tibial plateau fractures. The x-axis displays SHAP values (effect size on the model output); points are colored by feature value from blue (low) to red (high). Positive SHAP values indicate increased predicted risk. D-dimer and age are the dominant predictors, with higher values strongly associated with higher risk. Inflammatory markers (ESR, CRP) also show positive associations, supporting a role for inflammation in thrombogenesis. Lower PNI corresponds to higher risk, implicating compromised nutritional–immune status. Smoking and higher NEU increase risk, whereas lower LYM are likewise linked to higher risk. Figure 4B ranks features by mean absolute SHAP value; D-dimer, age, ESR, and PNI contribute most to risk prediction. Figure 4C illustrates feature-wise effects on predicted DVT risk. Higher D-dimer and age, together with lower LYM, correspond to larger SHAP values, indicating stronger positive contributions to risk. Notably, higher D-dimer is strongly associated with a marked increase in predicted DVT risk. By contrast, smoking and Schatzker type have smaller, relatively stable effects. NEU and PNI exhibit more complex patterns—NEU varies non-monotonically, whereas low PNI markedly increases risk. CRP and ESR rise with increasing values and then plateau, indicating pronounced non-linear effects. Taken together, these findings clarify each feature’s contribution to risk prediction and support clinical risk assessment.

**FIGURE 5:**
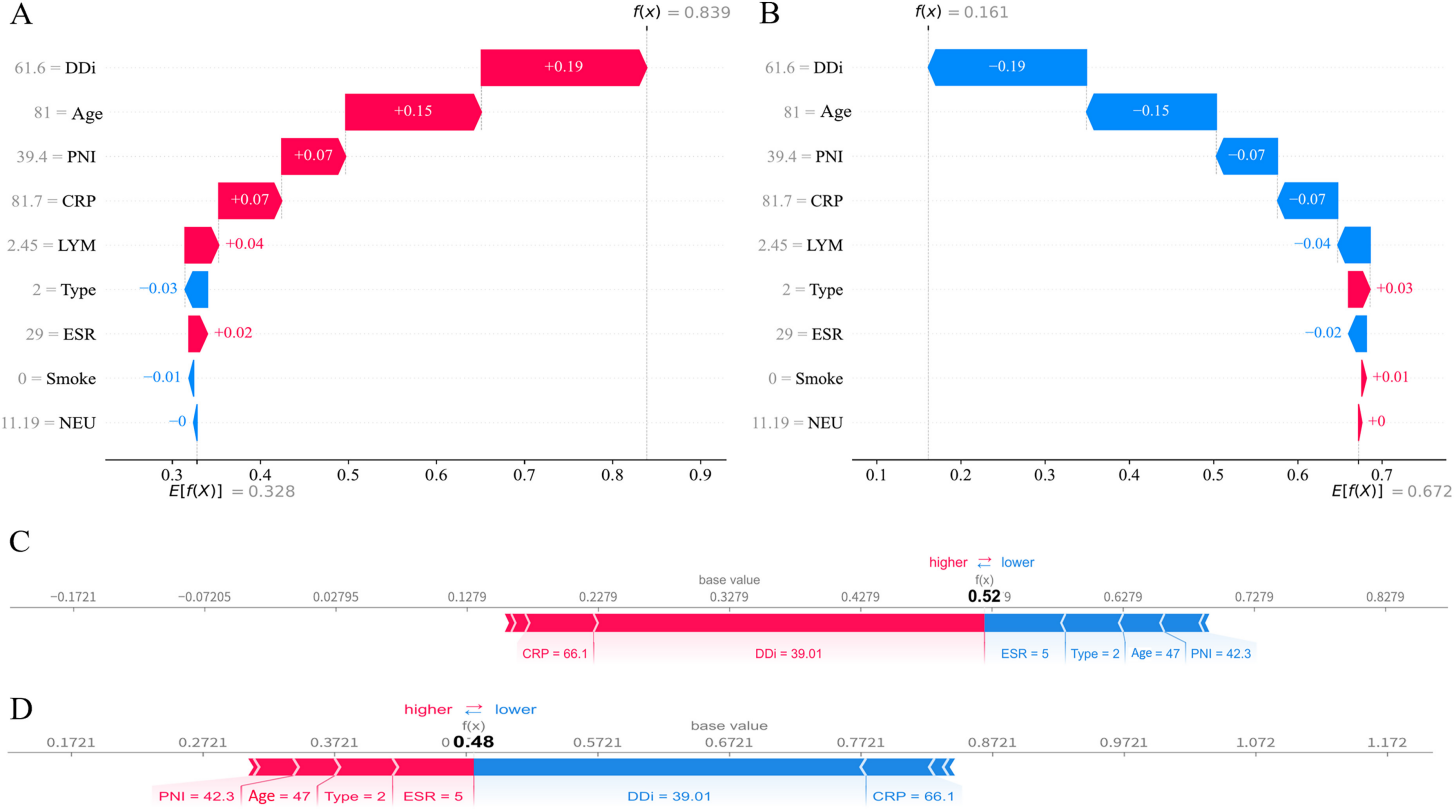
Local explanations of the extreme gradient boosting (XGBoost) model using Shapley Additive Explanations (SHAP). **(A,B)** Waterfall plots for Patient 1 under two outcome labels—**(A)** deep vein thrombosis (DVT) and **(B)** non-DVT—showing feature contributions shifting the prediction from the baseline expectation E[f(X)] to the patient-specific value f(x). **(C,D)** Force plots for Patient 2 under the same outcome labels—**(C)** DVT and **(D)** non-DVT. Positive SHAP values (red) increase the predicted probability of DVT; negative values (blue) decrease it.

Figures 5A,B display per-feature contributions (SHAP values) to the model output. Each line corresponds to one feature; blue bars indicate negative contributions to the predicted risk, and red bars indicate positive contributions. For the index patient, elevated D-dimer and older age—together with a low PNI—were the dominant positive shifts, yielding a final predicted probability of 0.839, consistent with high preoperative DVT risk. By contrast, smoking status and Schatzker type contributed little and were negative, slightly lowering risk. Figures 5C,D trace how each feature shifts the prediction from the model’s baseline to the final probability. Using the same covariates for the same patient, the plots contrast contribution profiles for the thrombotic versus non-thrombotic class. For example, in Figure 5C, a lower D-dimer than in the preceding patient reduced the model’s predicted probability, yielding a final value of 0.52 (i.e., a 52% preoperative DVT risk).

## Discussion

Using multidimensional preoperative features, we developed and compared several machine-learning models for preoperative DVT risk stratification in TPF. Balancing discrimination, calibration, and decision-curve performance, XGBoost emerged as the preferred model; by contrast, some tree-ensemble models showed training–validation performance gaps, suggesting overfitting. At the feature level, concordant selection by LASSO and Boruta, together with SHAP-based interpretability at global and individual levels, identified D-dimer, age, ESR, PNI, CRP, LYM, Schatzker type, NEU, and smoking as core predictors, underscoring the post-trauma coagulation–inflammation–nutrition axis and injury characteristics as key contributors to preoperative DVT risk.

Compared with prior studies, our work has three main strengths. First, population- and timing-specificity: most fracture-population DVT evidence centers on hip fractures or the postoperative phase; preoperative TPF data are limited, and heterogeneity stems from differences in screening strategies, test timing, and use of prophylactic anticoagulation. We explicitly anchored our study to the preoperative window and treated these factors as key contextual variables in both design and interpretation, improving comparability and external applicability. Second, multidimensional integration with interpretability: unlike prior TPF models that rely on traditional regression or single-dimension markers (e.g., nomograms based on SII or SIRI) (10, 11), which capture limited cross-domain interactions and non-linearity, we integrated injury features (Schatzker classification/laterality), temporal factors (injury-to-surgery interval), and coagulation–inflammation–nutrition indices (e.g., D-dimer, CRP/ESR, PNI) within a unified framework. Interpretable ML provided consistent global and individual-level explanations, aligning with recent trauma/arthroplasty work that combines ensemble learners (e.g., XGBoost) with explanation tools to enhance deployability (12–15). Third, clinically usable evidence: beyond discrimination, we reported calibration (Brier score, calibration curves) and decision-curve analysis, addressing a common gap in the literature and strengthening reproducibility and translational value.

Beyond these strengths, our machine-learning framework also offers several advantages over conventional regression-based approaches. In this study, logistic regression served as a benchmark model but, like other generalized linear models, it assumes linear and additive effects on the log-odds scale and requires prespecification of interaction and transformation terms. By contrast, the tree-based XGBoost algorithm can flexibly capture non-linear relationships and higher-order interactions among coagulation, inflammatory, nutritional–immune, and fracture-related variables without manual feature engineering. The SHAP dependence plots for D-dimer, ESR, CRP, NEU, and PNI illustrate plateauing or non-monotonic effects that would be difficult to parameterize within a traditional linear predictor. In addition, XGBoost achieved better overall discrimination and F1 performance than logistic regression on the validation set while maintaining acceptable calibration, suggesting greater suitability for risk stratification in this heterogeneous trauma population. Finally, coupling XGBoost with SHAP provided both global and patient-level explanations, allowing clinicians to visualize how individual predictors (e.g., D-dimer, age, PNI, smoking) jointly shift the predicted probability of preoperative DVT for a given patient—functionality that goes beyond the single-coefficient interpretation typically afforded by classical regression models.

Building on these strengths and the decision-curve analysis, we also outlined a simple preoperative clinical decision pathway to illustrate how the model could be incorporated into perioperative workflows (Supplementary Figure 1). In this framework, routinely available demographic, fracture, and laboratory variables (age, smoking status, Schatzker type, D-dimer, CRP, ESR, PNI, neutrophil and lymphocyte counts) are entered into the XGBoost model to generate an individualized predicted probability of preoperative DVT. Probability thresholds chosen within the range that yielded positive net benefit on decision-curve analysis can then be used to stratify patients into low-, intermediate-, and high-risk groups. Low-risk patients would generally continue to receive standard pharmacologic thromboprophylaxis with selective use of duplex ultrasonography according to symptoms and local resources; intermediate-risk patients could be prioritized for preoperative bilateral lower-limb duplex ultrasonography before definitive fixation; and high-risk patients could undergo prompt imaging with consideration of intensified prophylaxis and expedited operative planning. The specific cutoffs and management strategies would necessarily require local adaptation, external (including multicenter and temporal) validation, and prospective impact evaluation before being implemented in routine care, but this pathway highlights how a prediction model of this type might support ultrasound triage and perioperative decision-making.

The nine preoperative predictors identified in this study align with Virchow’s triad across multiple pathways—coagulation activation, inflammation–immune, nutrition–immune, and injury/exposure—and showed concordant evidence in explainability analyses. After intra-articular fracture, tissue-factor release and activation of the coagulation cascade drive fibrin generation and degradation; D-dimer therefore serves as a direct readout of trauma-related hypercoagulability. Our SHAP results identified D-dimer as the dominant feature, with a non-linear pattern characterized by a steep rise from low to mid ranges followed by a plateau, underscoring coagulation activation as central to risk stratification. In peri-knee and infragenicular fractures, admission D-dimer discriminates prevalent lower-extremity DVT and can support preoperative ultrasound triage (16, 17). Because baseline D-dimer increases with age, age-adjusted thresholds may improve ultrasound allocation in older adults (18); patients with elevated D-dimer should be prioritized for ultrasound or shortened rescreening intervals. Although trauma reduces D-dimer specificity, standardized sampling times and workflows preserve meaningful discrimination (19). Trauma-induced inflammation and thrombosis are mutually reinforcing. Systematic reviews and prospective studies link higher CRP to increased VTE risk (20, 21), and ESR—reflecting inflammatory burden—is positively associated with VTE in inflammatory phenotypes (22). In orthopedic trauma, neutrophil counts and derived ratios (NLR, SII, SIRI) correlate with perioperative DVT (23), potentially mediated by neutrophil extracellular traps, endothelial dysfunction, and thrombo-inflammatory crosstalk (24, 25). Consistent with this framework, our SHAP analysis highlighted CRP, ESR, and NEU as major contributors, supporting an “inflammation-driven hypercoagulability” model. Because PNI incorporates LYM, interpretation of LYM within the model should be linked to PNI to avoid substitution/interaction bias arising from structural correlation. As a composite of nutritional–immune reserve, PNI shows an independent inverse association with lower-extremity DVT in multicenter/large retrospective cohorts and adds diagnostic value beyond conventional risk factors (26), consistent with our finding that low PNI marks higher preoperative DVT risk. In preoperative orthopedic settings, hypoalbuminemia is an independent risk factor for preoperative DVT in hip/knee arthroplasty and patellar fractures (27, 28), suggesting that malnutrition may increase thrombotic susceptibility via inflammatory amplification and coagulation imbalance. Together, these data support perioperative assessment and optimization of nutritional and immune status to improve risk stratification and management in high-risk patients.

From an injury-biology perspective, Schatzker V–VI types often indicate greater articular depression/comminution and soft-tissue damage, with greater immobilization and inflammatory load, providing biologic plausibility for higher thrombotic susceptibility (29, 30). In a prospective cohort of 1,179 patients, V–VI types were associated with a higher proportion of preoperative DVT on univariable analysis; although variables such as open injury demonstrated stronger independence in multivariable models, this aligns with our inclusion of fracture type and its stable contribution (1). Basic and translational research shows that smoking promotes thrombosis through endothelial dysfunction, platelet activation, and pro-inflammatory pathways (31, 32); population-level data indicate a modestly higher VTE risk among current smokers (33), and orthopedic perioperative evidence likewise signals increased VTE complications with a smoking history (34). Persistent smoking should therefore be weighted in perioperative assessment and targeted for intervention, alongside fracture classification and soft-tissue status for individualized stratification. Age-related endothelial senescence, shifts in coagulation/fibrinolysis, and “inflammaging” shape a hypercoagulable phenotype; mechanistic studies implicate endothelial dysfunction, altered fibrin structure/function, and oxidative stress in venous thrombogenesis (35, 36). Clinically, age repeatedly associates with higher perioperative/trauma-related DVT risk across peri-knee and below-knee fractures. In a single-center ankle-fracture cohort, the preoperative DVT incidence was 6.4%, and each 10-year increase in age conferred significantly higher risk (37); retrospective work in intertrochanteric fractures likewise identified advanced age as an independent risk factor (38). In closed distal femur fractures, age ≥ 65 years together with elevated CRP indicated higher preoperative DVT risk (39), and a meta-analysis in hip fractures similarly identified advancing age as a consistent preoperative DVT predictor (40).

This study has several limitations. First, its single-center, retrospective design and reliance on one-time preoperative laboratory testing may introduce selection and information bias. Second, the outcome depended on preoperative duplex ultrasonography; variability in scan timing and operator performance could induce spectrum (and related verification) bias. Third, structural correlations among predictors may affect attribution of conditional effects in model interpretation. Fourth, we used a single-center sample with a stratified 70:30 holdout split and internal validation only; although we attempted to limit overfitting by combining this design with 5-fold cross-validation in the training set and by preferring models with smaller training–validation performance gaps, some degree of performance optimism and limited generalizability is unavoidable. Fifth, the cohort’s event rate and screening intensity may limit the transportability of threshold-dependent metrics and decision-curve net benefit to other settings; external application will require recalibration, local threshold setting, and preferably validation in independent cohorts. Finally, we did not incorporate quantitative imaging or perioperative time-series physiological features, and a prospective impact evaluation is still lacking.

## Conclusion

In patients with tibial plateau fractures, we developed and internally validated a preoperative DVT risk-stratification model using routinely available features in a single-center cohort. Considering discrimination, calibration, and decision-curve performance, XGBoost proved the most stable and interpretable among the candidate algorithms. The model centers on D-dimer, age, ESR, PNI, CRP, lymphocyte count, Schatzker type, neutrophil count, and smoking, outlining a biology in which coagulation–inflammation–nutrition axes interact with injury burden. It can support ultrasound triage and prioritize perioperative interventions. Before clinical deployment, multicenter and temporal external validation, recalibration with locally appropriate thresholds, and prospective impact assessment are needed to confirm benefits and safety for care pathways and outcomes.

## Data Availability

The original contributions presented in this study are included in this article/Supplementary material, further inquiries can be directed to the corresponding authors.

## Supplementary material

Supplementary Figure 1 Clinical decision pathway for preoperative deep vein thrombosis (DVT) risk stratification based on the XGBoost model.

Supplementary Figure 2 Pearson correlation heatmap of continuous predictors considered for model development.
