# Supplementary figures and images for: Preoperative deep vein thrombosis in tibial plateau fractures: development and internal validation of an interpretable multivariable machine-learning diagnostic model
Source: Front Med (Lausanne). 2026 Feb 13;13:1730477. doi: 10.3389/fmed.2026.1730477 (PMC12945751; doi:10.3389/fmed.2026.1730477)

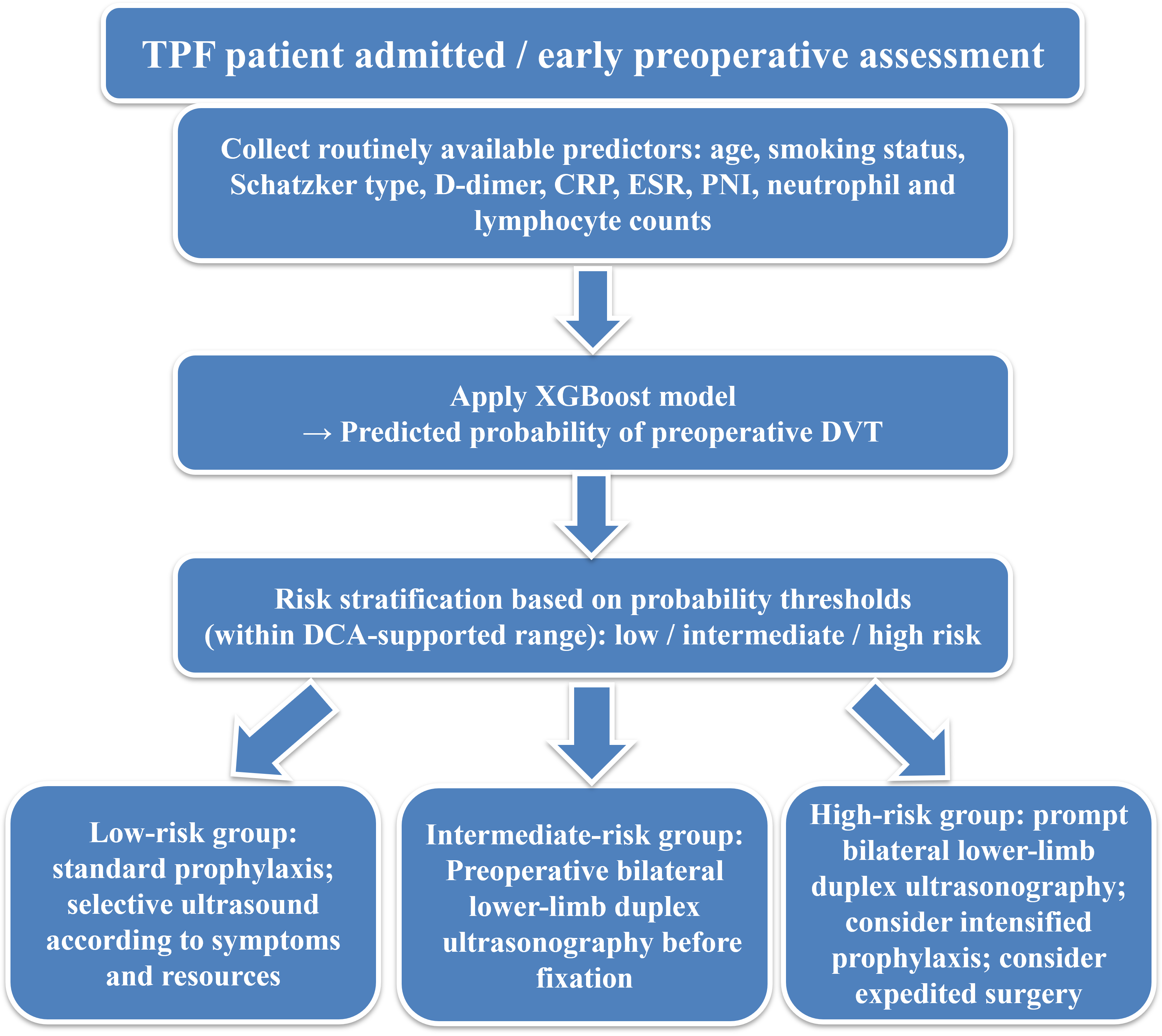

Supplement: Supplementary Figure 1 — Clinical decision pathway for preoperative deep vein thrombosis (DVT) risk stratification based on the XGBoost model. [file Image_1.tif]

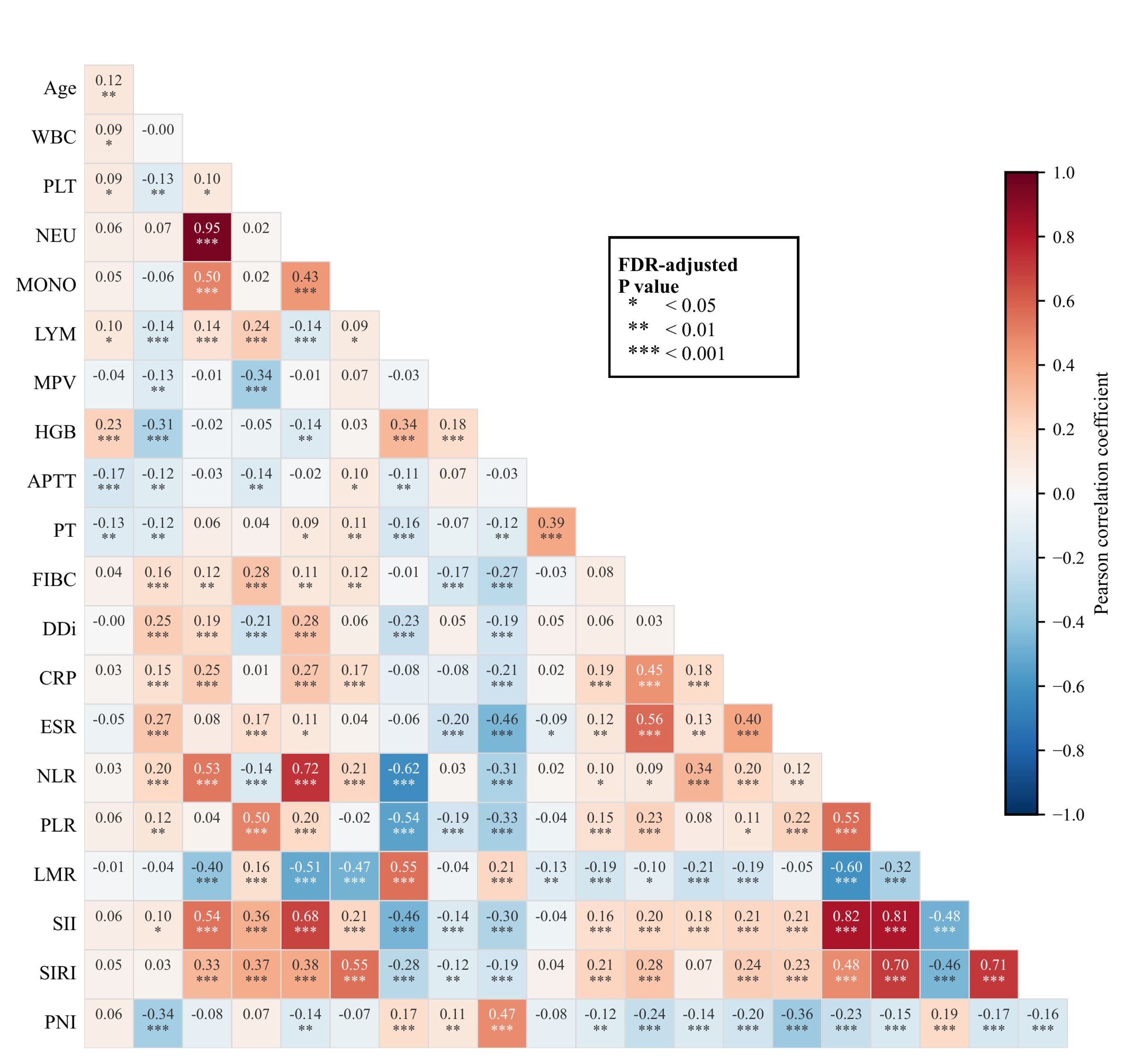

Supplement: Supplementary Figure 2 — Pearson correlation heatmap of continuous predictors considered for model development. [file Image_2.tif]
